# Butterfly in the Esophagus: A Case Report on a Rare Esophageal Manometry Catheter Malposition

**DOI:** 10.7759/cureus.32566

**Published:** 2022-12-15

**Authors:** Akshat Ritesh Shah, Maaz S Arif, We'am Hussain, Sangeeta Agrawal

**Affiliations:** 1 Gastroenterology, Dayton Veterans Affairs Medical Center, Wright State University, Dayton, USA

**Keywords:** high resolution manometry, dysphagia, butterfly, barium esophagram, reflux esophagitis

## Abstract

We report the case of a 75-year-old man who underwent high-resolution manometry (HRM) testing for solid food dysphagia after an unrevealing upper endoscopy and biopsies. A barium esophagogram confirmed nonspecific motility disorder. A subsequent HRM study was performed, but when all swallow studies were noted to fail, and the manometric images revealed a *butterfly wings* appearance, it was found that the manometry catheter was actually coiled and folded back cephalad. As there are only a few other case reports with similar presentations, we believe this case would serve as a good reminder for clinicians to practice caution when cannulating the manometry catheter.

## Introduction

Despite its seemingly simple nature, the human swallow is a complex neuromuscular function divided into several phases involving multiple muscles to coordinate synchronously. Therefore, any interruptions in this multistage process can lead to dysfunction or difficulties in swallowing, known as dysphagia. Acute dysphagia results from the inability to swallow solids or liquids and requires immediate attention. One modality used to evaluate nonobstructive esophageal dysphagia is manometry, in which an esophageal manometry catheter is placed in a patient's nares and the patient is asked to take 10 swallows with subsequent measurements of esophageal pressures. The first manometric systems were developed in the 1970s. High-resolution manometry (HRM), invented in the 1990s, allowed for a shorter procedure duration, increased pressure sensing quality, and simultaneous monitoring of the upper esophageal sphincter, esophagus, and lower esophageal sphincter [1]. Even though HRM has improved the diagnostic accuracy of esophageal motility disorders, it is not devoid of technical imperfections. A prevalence rate of 21% for technically imperfect studies has been previously reported for HRM studies [2]. Traversing the esophagogastric junction (EGJ) with the HRM catheter can be particularly challenging, resulting in imperfect studies. The coiling of the HRM catheter at the EGJ results in the formation of a butterfly pattern as the pressure waves appear symmetrical below and above the presumed EGJ location. Here, we report a case of a 75-year-old man in whom the HRM study revealed a butterfly pattern due to the coiling of the manometric catheter at the EGJ.

## Case presentation

A 75-year-old man presented to the clinic for HRM for the evaluation of dysphagia. He reported a long-standing history of dysphagia to predominantly solid food, associated with vomiting, with symptoms progressively worsening with time. He denied any weight loss, fevers, cough, or a family history of gastrointestinal malignancy. Vital signs were normal, and physical examination did not reveal any abdominal tenderness or distension. He initially underwent an upper endoscopy months ago that was unrevealing for any intraluminal mass/abnormalities, and esophageal biopsies were significant for mild reflux esophagitis but negative for dysplasia or eosinophilic esophagitis (EoE). An empiric dilation with a 48 French Savary dilator (Savary-Gilliard® Dilator, Bloomington, IN, USA) did not provide symptomatic improvement.

Afterward, a barium esophagram revealed abnormal peristaltic waves indicative of a nonspecific esophageal motility disorder. Subsequent esophageal manometry revealed that all swallows failed peristalsis and the absence of any contractility (Table 1). 

**Table 1:** Manometry analysis report demonstrating failure of evaluated 10 swallows with no effective peristalsis or contractions.

Esophageal motility	
Number of swallows evaluated	10
Chicago classification	
Failed (%)	100
Weak (%)	0
Ineffective (%)	100
Pan-esophageal pressurization (%)	100
Premature contraction (%)	0
Rapid contraction (%)	0
Fragmented (%)	0
Intact (%)	0
Number of hypercontractile swallows	0

**Figure 1:**
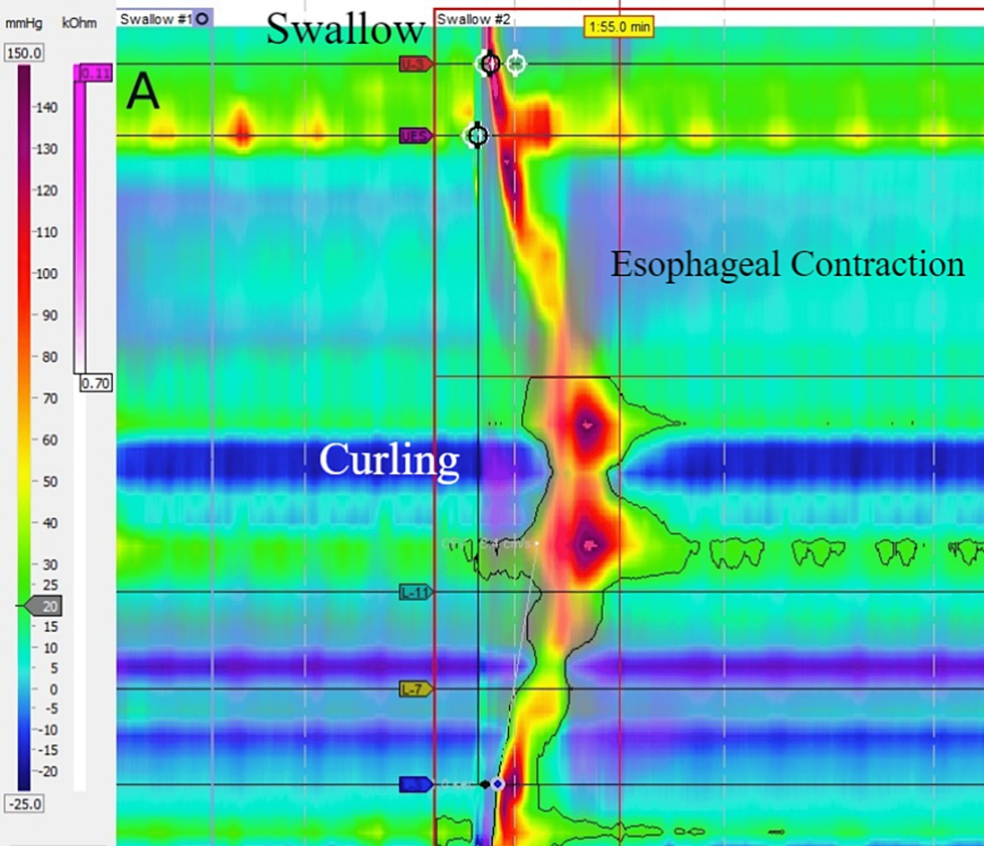
High-resolution manometry of one swallow revealing the characteristic butterfly wings effect, or mirror image, with two closely fused pressure zones and symmetric peristalsis, suggestive of a cephalad folded and coiled manometry catheter in the esophagus.

The images show the characteristic *butterfly *wings effect, or mirror image (Figure 1).

Because the catheter was placed blindly, its malposition was noted in the report days after the procedure. The patient was contacted to explain the error and repeat the study in the near future.

## Discussion

Dysphagia, defined as the sensation of the impaired passage of food from the mouth to the stomach, is a significant problem in the elderly [3]. Clinically, it is classified into two distinct types, oropharyngeal and esophageal, mostly distinguishable based on the patient's history. Oropharyngeal dysphagia is characterized by difficulty swallowing accompanied by deglutitive postnasal regurgitation, coughing, drooling, or aspirations. Esophageal dysphagia is characterized by a delayed sensation of food sticking in the upper or lower chest [4]. Various investigations, including upper endoscopy, barium radiography, radionuclide transit studies, and manometry, may be used to evaluate esophageal dysphagia, with each providing somewhat different information. However, esophageal manometry is the test of choice for evaluating esophageal motor function [5].

Esophageal manometry assesses esophageal motility patterns by measuring the amplitude of contractile events in the esophagus and its sphincters in relation to time. The introduction of practical HRM systems and the development of an advanced software algorithm that converts HRM data to seamless and dynamic spatiotemporal esophageal pressure topography (EPT) have revolutionized the performance of clinical esophageal manometry [6]. HRM uses up to 36 pressure sensors, each spaced 1 cm apart along a catheter, with the distal sensor positioned 2 to 3 cm below the diaphragm. The data from these sensors is then converted to EPT. This multitude of closely spaced sensors eliminates the problem of movement-related artifacts, a major weakness of conventional manometric systems [7]. With HRM data and EPT, it soon became apparent that many esophageal motility disorders could be recognized as distinct patterns, which led to the introduction of a new classification for the esophageal motility disorders known as the Chicago classification [5]. In addition, various studies comparing conventional line tracing and HRM have reported improved diagnostic accuracy, ease of interpretation, and better interobserver agreement with HRM [6].

A standard HRM protocol includes a 30-second baseline recording without swallowing followed by ten 5 mL, room temperature water swallows in the supine position. The baseline motility pattern provides information regarding anatomic profiles and resting pressures, while the motility patterns during swallowing provide valuable information about esophageal contractility and sphincter relaxation in response to bolus [6]. Within the baseline window, the pressure inversion point (PIP) - the point of transition from the intraabdominal cavity to the intrathoracic cavity - is identified. PIP identification is important because its presence indicates that the EGJ was traversed during the procedure.

Technical imperfections are frequently encountered while performing HRM studies. In a review of 2,000 consecutive EPT studies, Roman et al. [2] reported a prevalence of 21% for technically imperfect studies, although most were minor imperfections. The most frequently encountered technical limitation (12%) was incomplete swallow protocol, defined as fewer than seven analyzable swallows. The second most common limitation (6%) was the inability to traverse the EGJ. Technical malfunctions (sensor problem and absence of thermal compensation) and recording artifacts were encountered infrequently in <1.5% of cases each. Even with all these technical limitations, the diagnosis of achalasia was correctly made in 94% of cases, similar to that achieved with perfect studies. Only 6.5% (27 cases) of all the technically imperfect studies were judged nondiagnostic and required further workup. The most common cause of nondiagnostic studies was the inability to traverse the EGJ junction [2].

Traversing the EGJ with the HRM catheter can be challenging, and a keen observation by the operator should be made to ascertain the optimal placement of the catheter. A simple deep inspiration maneuver may help localize the diaphragm as the pressure goes up in the abdomen and down in the chest with inspiration [5]. Lack of passage across the EGJ is suspected when the PIP is absent and/or a butterfly pattern of recurring pressure bands is encountered (pressure waves appear symmetrical below and above the presumed EGJ location). The most frequently reported causes of inability to traverse EGJ were hiatal hernia (50%), achalasia (24%), previous foregut surgeries noted in 17% of cases (fundoplication, gastric bypass, gastrectomy, esophagectomy, and gastric stapling). Other miscellaneous causes were small hiatal hernia, extreme angulation at the EGJ, distal stricture, and narrowing of the esophagus due to eosinophilic esophagitis, scleroderma, neoplasia of the tongue, and dilated esophagus on the chest X-ray [2].

Clinical maneuvers like change in the patient’s position (standing up and overhead hand raise), repeated sips of water to relax the EGJ junction, and 45° to 90° clock or counterclockwise rotation along the long axis of the catheter to reposition may be tried in these instances. Endoscopic guided catheter placement may also be tried on the failure of these maneuvers. A success rate of 90% has been reported for endoscopic placement in achalasia patients compared to 36% in patients with large hiatal hernias [8]. Due to the high failure rate in patients with large hiatal hernias, Kim [9] developed a new catheter with an optical module in the front to provide forward images and better visibility during catheter insertion. This vision-assisted manometry catheter offers a real-time snapshot of the catheter position and can be used as an alternative for endoscopic placement [9].

Our patient's barium esophagogram revealed a nonspecific esophageal motility disorder; a subsequent esophageal manometry revealed failed peristalsis and the absence of contractility. Further review of the spatiotemporal manometric representations revealed the *butterfly* pattern, suggesting the lack of passage across the EGJ. As we were not privy to catheter mispositioning during blind placement until after the report was generated days after, the patient was contacted for a repeat study.

## Conclusions

In conclusion, esophageal manometry is an important aspect in the evaluation of nonobstructive esophageal dysphagia. We report a rare case of an improperly coiled esophageal manometric catheter that resulted in a characteristic *butterfly*, or mirror image. If possible, any aberrances in catheter positioning should be recognized and corrected appropriately by the operator. While no definitive diagnosis was able to be made, the patient was instructed to follow up on an outpatient basis.
